# Diaphragm dysfunction, lung aeration loss and weaning-induced pulmonary oedema in difficult-to-wean patients

**DOI:** 10.1186/s13613-021-00886-6

**Published:** 2021-06-28

**Authors:** Martin Dres, Emmanuel Rozenberg, Elise Morawiec, Julien Mayaux, Julie Delemazure, Thomas Similowski, Alexandre Demoule

**Affiliations:** 1 Service de Pneumologie, Médecine intensive – Réanimation (Département “R3S”), AP-HP. Sorbonne Université, Hôpital Pitié-Salpêtrière, 75013 Paris, France; 2 Sorbonne Université, INSERM, UMRS1158 Neurophysiologie Respiratoire Expérimentale et Clinique, Paris, France

**Keywords:** Diaphragm, Pulmonary oedema, Difficult weaning, Lung ultrasound

## Abstract

**Background:**

Diaphragm dysfunction and weaning-induced pulmonary oedema are commonly involved during weaning failure, but their physiological interactions have been poorly reported. Our hypothesis was that diaphragm dysfunction is not particularly associated with weaning-induced pulmonary oedema.

**Methods:**

It was a single-centre and physiological study conducted in patients who had failed a first spontaneous breathing trial and who underwent a second trial. The diaphragm function was evaluated by measuring the tracheal pressure generated in response to a bilateral magnetic phrenic nerves stimulations. Weaning-induced pulmonary oedema was diagnosed in case of failure of the spontaneous breathing trial if patients exhibited signs of plasma concentration or echocardiographic diagnosis of pulmonary artery occlusion pressure elevation.

**Results:**

Fifty-three patients were included and 31/53 (58%) failed the spontaneous breathing trial, including 24/31 (77%) patients with weaning-induced pulmonary oedema. Diaphragm dysfunction was present in 33/53 (62%) patients. Diaphragm dysfunction or weaning-induced pulmonary oedema were present in 26/31 (84%) of the patients who failed the spontaneous breathing trial. Weaning-induced pulmonary oedema occurred in 20/33 (61%) patients with a diaphragm dysfunction and in 4/20 (20%) patients without (*p* = 0.005).

**Conclusion:**

Weaning-induced pulmonary oedema was three times more frequent in case of diaphragm dysfunction. Even in case of diaphragm dysfunction, physicians might be encouraged to investigate the presence of weaning-induced pulmonary oedema during weaning failure.

## Introduction

Weaning-induced pulmonary oedema is a frequent cause of spontaneous breathing trial failure, accounting for up to 60% of weaning failure in two large observational studies [1, 2]. Likewise, a strong association has been established between diaphragm dysfunction and spontaneous breathing trial failure [3, 4]. While the corresponding mechanisms of diaphragm dysfunction and spontaneous breathing trial failure remain incompletely elucidated, they are likely to involve a combination of reduced diaphragm strength [5] and increased lung impedance [6] due to atelectasis secondary to diaphragm weakness [7–9] leading to respiratory load–capacity imbalance. Although diaphragm dysfunction and weaning-induced pulmonary oedema are two important causes of spontaneous breathing trial failure, no study has ever investigated their interaction. On the one hand, diaphragm dysfunction reduces the rise in thoraco-abdominal gradient that could potentially decrease the risk of weaning-induced pulmonary oedema [10, 11]. On the other hand, the respiratory distress that occurs during a failed spontaneous breathing trial involves large negative swings in pleural pressure that influence cardiac performance by increasing the venous return and/or increasing left ventricular afterload [12]. It may result in turn in weaning-induced pulmonary oedema [13, 14]. In theory, a weak diaphragm should lessen the haemodynamic consequences of spontaneous ventilation during respiratory distress. In addition, diaphragm dysfunction may also cause lung aeration loss that could subsequently lead to weaning failure [15]. We hypothesized that diaphragm dysfunction would be more likely associated with lung aeration loss than with weaning-induced pulmonary oedema. The first objective was to describe the incidence of weaning-induced pulmonary oedema in patients with or without diaphragm dysfunction. Secondary objective was to compare the lung aeration loss according to the presence or absence of diaphragm function.

## Methods

This study complies with the Strengthening the Reporting of Observational Studies in Epidemiology (STROBE) Statement. It was conducted in a medical intensive care unit over a 14-month period between April 2018 and June 2019. The study was approved by the Comité de Protection des Personnes du Sud Ouest et Outre Mer 4 (RCB ID: 2018-A00176-49). Written and oral information about the study was given to patients or their families prior enrolment. Informed consent was obtained from all patients or their relatives.

### Study population

Patients older than 18 years, ventilated via an endotracheal tube for more than 48 h and who had already failed a first spontaneous breathing trial were eligible. We sought to enrol patients early during the weaning process as soon as the readiness-to-wean criteria (see below) were present. Patients undergoing spontaneous breathing trial during holidays and weekends were not considered for inclusion. Patients had to meet predefined readiness-to-wean criteria on daily screening: SpO_2_ > 90% or PaO_2_/FiO_2_ ≥ 150 mmHg with a fraction of inspired oxygen (FiO_2_) ≤ 40% and a positive end-expiratory pressure ≤ 8 cmH_2_O] [16]. In our practice, readiness-to-wean criteria are sought for to conduct a spontaneous breathing trial regardless of the presence of readiness to extubate criteria which are the following: cough strength, neurological status and abundance of secretions. Therefore, In case of successful spontaneous breathing trial, extubation may not be considered. Pregnant women, patients with contraindications to magnetic phrenic nerve stimulation (chest tube, cardiac pacemaker or implanted defibrillator, cervical implants), and patients in whom weaning was impossible (pre-existing neuromuscular disorders, cervical cord injury) were not considered for inclusion.

### Measurements and data collection

Upon inclusion, demographic data were prospectively collected: age, gender, comorbidities (chronic hypertension, chronic obstructive pulmonary disease, chronic left ventricle failure), sequential organ failure assessment and simplified acute physiology score, date of intensive care unit admission, date of intubation, main reason for intubation, weight and height upon admission and fluid balance over the last 24 h. In addition, the following clinical variables were prospectively collected before and at the end of the spontaneous breathing trial: systolic and diastolic arterial pressure, heart rate, respiratory rate, SpO_2_. Arterial blood gases, lactate, plasma protein concentration and haemoglobin were sampled before and at the end of the spontaneous breathing trial. Haemoglobin concentration was measured from the arterial blood sample by the ABL 800flex device (Radiometer, Copenhagen, Denmark). The repeatability of the haemoglobin concentration measurements with the ABL 800flex is reported by the manufacturer to be 0.6% ± 1.4%. The plasma protein concentration was measured with the Cobas 7000 c501 device (Roche Diagnostics, Basel, Switzerland). The repeatability of plasma protein concentration measurements with this device is reported by the manufacturer to be 0.6% ± 0.9%.

### Diaphragm function evaluation

Before conducting the spontaneous breathing trial, diaphragm function was assessed in terms of the changes in endotracheal tube pressure induced by bilateral phrenic nerve magnetic stimulation during airway occlusion (Ptr, stim), as described in other reports [3]. Briefly, two figure-of-eight coils connected to a pair of Magstim® 200 stimulators (The Magstim Company, Dyfed, UK) were positioned immediately posterior to the sternomastoid muscles at the level of the cricoid cartilage. Bilateral phrenic nerve stimulation was performed while the endotracheal tube was manually occluded and stimulations were delivered at the maximum intensity allowed by the stimulator (100%) known to result in supramaximal diaphragm contraction [3, 17]. The patients were studied in a standardized semi-recumbent position during a brief disconnection of the endotracheal tube from the ventilator. While the endotracheal tube was manually occluded, bilateral anterolateral magnetic stimulation was performed. The absence of active respiratory efforts was verified by checking the absence of drop in airway pressure signal on the laptop’s screen. Two operators (MD, ER) were required to achieve both stimulation and measurements. After positioning the coils, at least three stimulations were performed at 100% of maximal output allowed by the stimulator. Stimulations were separated by at least 60-s to avoid superposition. The average of three measures was taken into account for analysis. Ptr,stim was defined as the amplitude of the negative pressure wave following stimulation, taken from baseline to peak. It was measured at the proximal external end of the endotracheal tube, using a linear differential pressure transducer (MP45 ± 100 cmH_2_O, Validyne, Northridge, Calif., USA). The pressure signal was sampled and digitized at 100 Hz (MP30, Biopac Systems, Santa Barbara, Calif., USA or Powerlab, AD Instruments, Bella Vista, Australia) for subsequent data analysis. Diaphragm function assessment was repeated at the end of the spontaneous breathing trial in the absence of sustained respiratory distress at the end of the spontaneous breathing trial (SpO2 < 90%, respiratory rate > 30/min for a least 5 min).

### Cardiac function evaluation

Cardiac function was evaluated before conducting the spontaneous breathing trial by transthoracic echocardiography equipped with a cardiac probe (Sparq, Philips) performed by a fully trained and experienced operator (ER) and reviewed off-line by a second operator (MD). The following variables were obtained from the apical four-chamber view: left ventricular ejection fraction (visual estimation), early (E) and late (A) diastolic wave velocities at the mitral valve, tissue Doppler early (e’) wave velocity at the lateral mitral valve annulus, the deceleration time of the E wave, cardiac output as estimated by the stroke volume measured using the Doppler method applied at the left ventricular outflow tract, tricuspid annular plane systolic excursion in M-mode, peak systolic velocity at the lateral tricuspid annulus and systolic pulmonary arterial pressure estimated by the tricuspid regurgitation maximal velocity pressure gradient. Finally, colour Doppler mapping was used to detect the presence of significant mitral regurgitation and semiquantitatively assess its severity. The cardiac assessment was completed by a 12-lead electrocardiogram.

### Lung aeration

Lung ultrasound (Philips Sparq) was performed by a trained investigator, who acquired all images of the study. As previously described [15], a 2–4 MHz convex probe was used to scan the whole lung on both sides. The number of B-lines was counted on a rib short-axis scan between two ribs at each intercostal space of the upper and lower parts of the anterior, lateral, and posterior regions of the left and right chest wall (total of 12 areas). For a given region of interest, points were allocated according to the worst ultrasound pattern observed: presence of lung sliding with A lines or fewer than two isolated B lines = 0, moderate loss of lung aeration: multiple, well-defined B lines = 1, severe loss of lung aeration: multiple coalescent B lines = 2, lung consolidation = 3 [15]. Skin was marked to apply the probe at precisely the same area before and after the spontaneous breathing trial. In addition to lung ultrasound, the presence of pleural effusion was also investigated and evaluated, as previously described (small, moderate, large) [18].

### Study design

Patients eligible to the study underwent a spontaneous breathing trial. The spontaneous breathing trial was performed for 30 min (or less in case of clinical intolerance) with the pressure support level and positive end-expiratory pressure set to zero (so called 0–0 test), while FiO_2_ remained unchanged. This spontaneous breathing trial modality is similar to the “T-tube” and reflects equally the work of breathing after extubation [20]. Spontaneous breathing trial failure was defined by the following criteria: respiratory rate ≥ 35 breaths/min or increase ≥ 50%, SpO_2_ ≤ 90% or PaO_2_ ≤ 50 mmHg (with FiO_2_ ≥ 50%), heart rate ≥ 140 bpm, de novo supraventricular or ventricular arrhythmia, systolic arterial pressure > 180 or < 90 mmHg, alteration of consciousness, and diaphoresis or any signs of respiratory distress [16]. In the event of spontaneous breathing trial failure, the pre-spontaneous breathing trial settings were resumed.

After obtaining consent and before starting the spontaneous breathing trial, clinical and laboratory variables were collected. Electrocardiogram, echocardiography, lung ultrasound and diaphragm evaluation were performed. The spontaneous breathing trial was then started. At the end of the spontaneous breathing trial or sooner in case of clinical intolerance, electrocardiogram, echocardiography and lung ultrasound were repeated before resuming the initial ventilator settings.

### Definitions

Diaphragm dysfunction was defined by a Ptr,stim below 7 cmH_2_O [21–23]. Weaning-induced pulmonary oedema was defined in case of spontaneous breathing trial failure associated with at least one of the following two features:(1) echocardiographic diagnosis of pulmonary artery occlusion pressure elevation (E/A ratio above 0.95 and E/e’ ratio above 8.5 during the spontaneous breathing trial [24]) and/or (2) plasma protein concentration (5% increase in haemoglobin and/or plasma protein concentration) during the spontaneous breathing trial [25, 26].

### Statistical analysis

Data were expressed as median (interquartile range) or number (%). Comparisons between spontaneous breathing trial success and spontaneous breathing trial failure were assessed with a Mann–Whitney U test. Baseline Ptr,stim were compared between patients with spontaneous breathing trial success and patients with spontaneous breathing trial failure associated with or without weaning-induced pulmonary oedema with a Kruskal–Wallis test. Baseline lung ultrasound score was compared between patients with spontaneous breathing trial success and patients with spontaneous breathing trial failure and the presence or absence of diaphragm dysfunction with a Kruskal–Wallis test. The correlation between Ptr,stim and lung ultrasound score was assessed with the non-parametric Spearman correlation. The proportion of patients with weaning-induced pulmonary oedema and with spontaneous breathing trial failure for another reason was compared with a Fisher’s exact test. A convenient sample size was defined on the basis of previous similar physiological investigations [25, 27, 28]. Regarding the characteristics of the population included, we assumed a spontaneous breathing trial success/failure rate of 50%/50% [1, 25] and planned to include a minimum of 25 patients per group. We also expected a dropout rate of approximately 10% due to technical problems, and therefore planned to enrol 55 patients. For all final comparisons, a p value less than or equal to 0.05 was considered to be statistically significant. Analyses were performed using Prism 8.3.0 software (GraphPad Software, USA).

## Results

### Study population

**Fig. 1:**
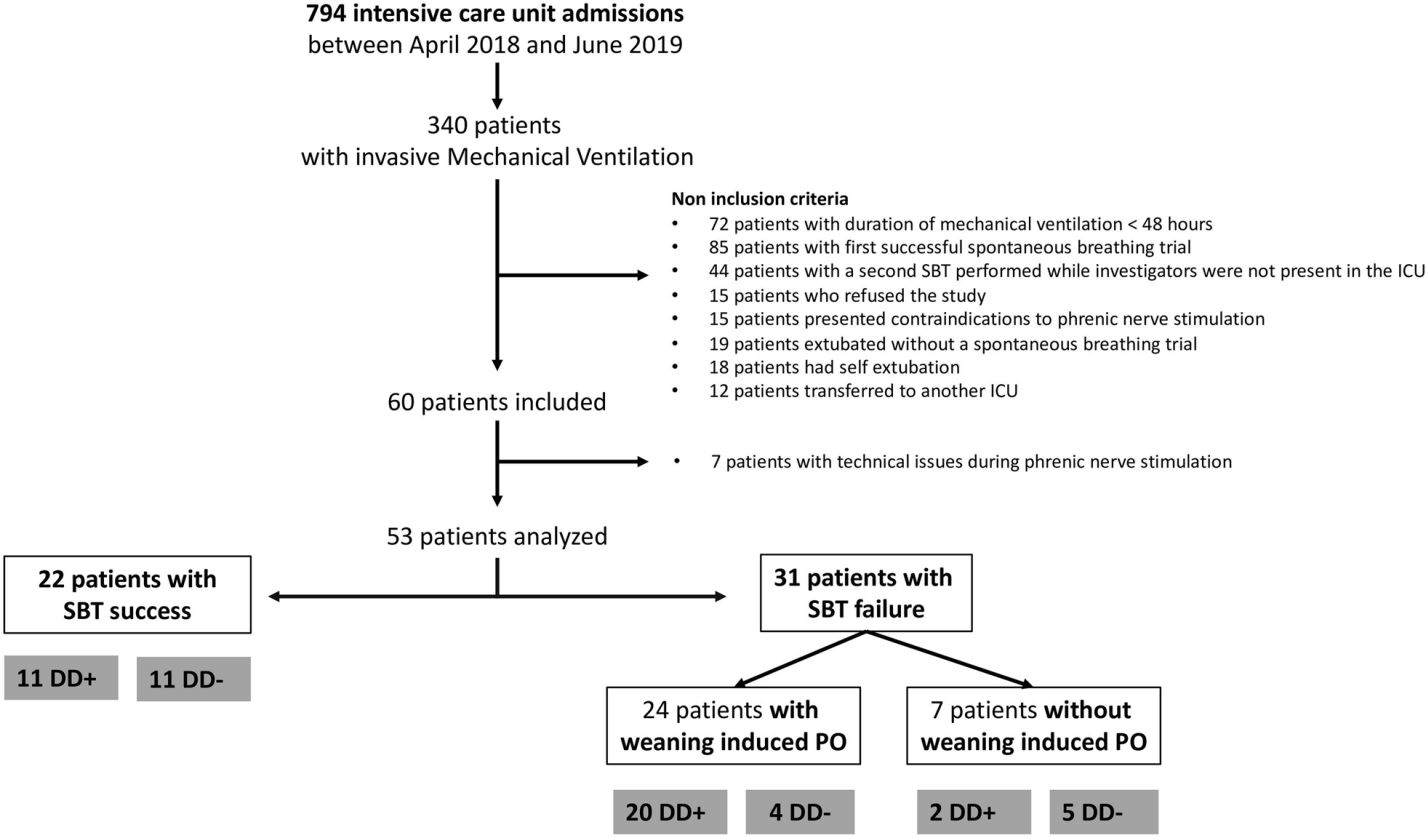
Study flowchart. ICU: intensive care unit; SBT: spontaneous breathing trial; PO: pulmonary oedema; DD: diaphragm dysfunction

During the study period, 794 patients were admitted to the ICU and 340 received invasive mechanical ventilation. Sixty of these patients were enrolled, seven of whom were subsequently excluded for technical reasons (Fig. 1). Overall, 53 patients were analysed. The main reason for intubation was hypoxemic acute respiratory failure and the median SAPS II score was 61 (49–76). At the time of inclusion, the median duration of mechanical ventilation was 4 (2–9) days (Table 1) and all patients were ventilated with pressure support mode. Overall, left ventricular ejection fraction was 60% (50–60) and less than 50% in 5 patients. No significant valvular regurgitation, hypertrophic cardiomyopathy or pericardial effusion was documented. Atrial fibrillation was present in three patients. Thirteen patients had a small-to-moderate pleural effusion. Diaphragm dysfunction was demonstrated in 33 (63%) patients and median Ptr,stim was 4.7 (2.9–8.4) cmH_2_O.

**Table 1:** Baseline patient characteristics

	SBT success *N* = 22	SBT failure *N* = 31	*p*
Age, years	61 (50–71)	63 (55–63)	0.638
Female, *n* (%)	10 (45)	15 (48)	0.999
Body mass index, kg.m^−2^	24 (21–27)	25 (20–30)	0.609
Pre-existing conditions, *n* (%)			
Hypertension	8 (36)	10 (32)	0.776
Chronic left ventricle failure	4 (18)	8 (26)	0.740
COPD	3 (14)	4 (13)	0.999
SAPS 2	57 (47–77)	65 (49–76)	0.493
SOFA	10 (6–11)	10 (7–12)	0.756
Fluid balance over last 24 h, L	− 0.6 (− 1.2–0.7)	− 0.8 (− 1.6–0.7)	0.205
Length of ICU stay, days	5 (2–6)	5 (2–12)	0.570
Duration of mechanical ventilation, days	4 (2–6)	5 (2–11)	0.468
SBT before inclusion, *n*	1 (1–1)	1 (1–1)	0.113
Main reason for intubation, *n* (%)			
Acute respiratory failure	13 (59)	16 (52)	0.779
Coma	7 (32)	12 (39)	0.772
Shock	1 (5)	1 (3)	0.999
Post-surgery	1 (5)	2 (6)	0.999
Ventilator settings			
Pressure support, cmH_2_O	10 (7–10)	12 (10–12)	< .001
PEEP, cmH_2_O	5 (5–6)	6 (5–6)	0.076
Expired tidal volume, ml.kg^−1^ IBW	7 (7–9)	7 (6–7)	0.053

SBT, spontaneous breathing trial; COPD, chronic obstructive pulmonary disease; SAPS, Simplified Acute Physiology Score; SOFA Sequential Organ Failure Assessment; ICU, intensive care unit; PEEP, positive end-expiratory pressure; IBW, ideal body weight

### Reasons for spontaneous breathing trial failure

**Fig. 2:**
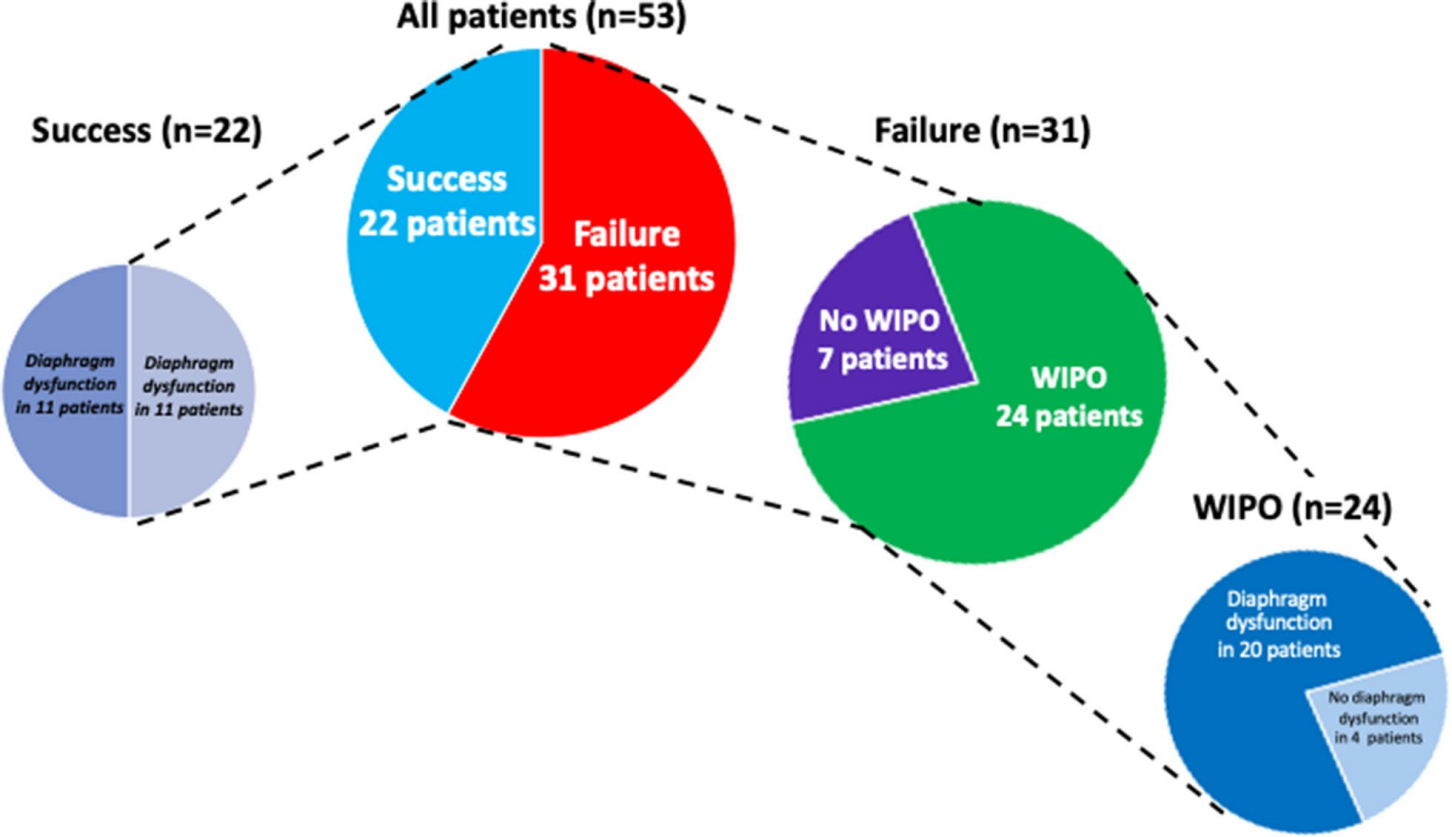
Outcomes of the spontaneous breathing trial presented with the proportion of patients with diaphragm dysfunction and weaning-induced pulmonary oedema (WIPO)

Thirty-one (58%) of the 53 patients enrolled in the study failed the spontaneous breathing trial. Weaning-induced pulmonary oedema was documented in 24 (77%) of these 31 patients (Figs. 1 and 2). Among the 24 patients with weaning-induced pulmonary oedema, 20/24 had an increase in plasma protein concentration or in haemoglobin or both, 15/24 had an increase in E/A and E/e’ ratios and 11/24 had a combination of ultrasound indices and biomarkers.

Among the 31 patients who failed the spontaneous breathing trial, 20 (83%) of the 24 patients with documented weaning-induced pulmonary oedema had diaphragm dysfunction versus 2 (29%) of the 7 patients who failed the spontaneous breathing trial for other reasons (*p* = 0.012) (Fig. 2). Of the seven patients who failed the spontaneous breathing trial for a reason other than weaning-induced pulmonary oedema, two patients failed because of copious secretions, one patient developed central hypoventilation due to Arnold–Chiari malformation, and no precise reason was found in the four remaining patients. Of note, diaphragm dysfunction was present in two of these four patients (Ptr,stim was −2.7 cmH_2_O and −3.0 cmH_2_O). Overall, diaphragm dysfunction or weaning-induced pulmonary oedema was present in 26/31 (84%) of the patients who failed the spontaneous breathing trial.

### Impact of diaphragm function on cardiac function during the spontaneous breathing trial

**Fig. 3:**
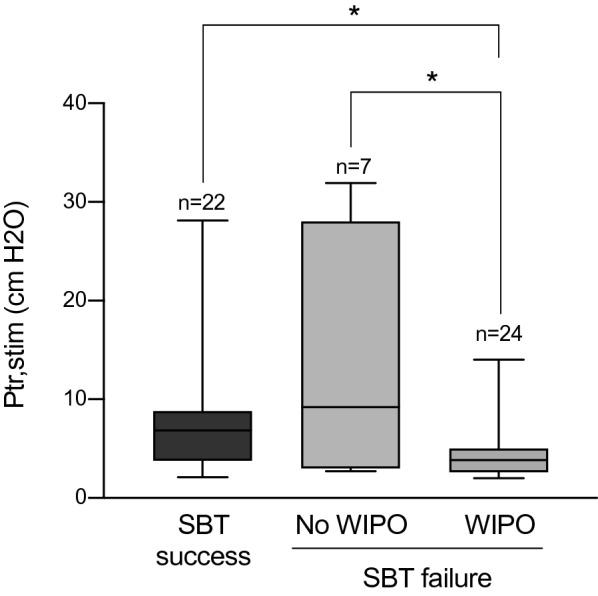
Diaphragm function as evaluated by measuring the change in tracheal pressure in response to bilateral phrenic nerve stimulation (Ptr,stim) in patients with a successful spontaneous breathing trial (SBT) and those with SBT failure with and without weaning-induced pulmonary oedema (WIPO). Box plots show the median (interquartile)

Weaning-induced pulmonary oedema occurred in 20/33 (61%) patients with a diaphragm dysfunction and in 4/20 (20%) patients without (*p* = 0.005). Clinical variables, echocardiographic measurements and diaphragm function before and at the end of the spontaneous breathing trial are presented in Table 2. Ptr,stim was higher in patients who failed the spontaneous breathing trial for reasons other than weaning-induced pulmonary oedema compared to those who failed the spontaneous breathing trial because of weaning-induced pulmonary oedema (Fig. 3). No correlation was found between Ptr,stim and the changes in protein, haemoglobin, E/A ratio and E/e’ ratio during the spontaneous breathing trial.

**Table 2:** Clinical variables, gas exchange, cardiac function and lung aeration before and at the end of the spontaneous breathing trial

	Before the SBT			End of the SBT		
	SBT success	SBT failure	*p*	SBT success	SBT failure	*p*
*n*	22	31		22	31	
Clinical variables						
Systolic ABP, mmHg	121 (110–139)	140 (118–151)	0.041	130 (109–154)	149 (128–170)	0.007
Heat rate, min^−1^	94 (77–101)	93 (84–104)	0.840	97 (86–105)	105 (96–121)	0.036
Respiratory rate, min^−1^	19 (15–23)	21 (18–26)	0.101	21 (19–31)	28 (21–35)	0.030
Laboratory variables						
pH	7.42 (7.38–7.48)	7.43 (7.38–7.45)	0.732	7.43 (7.40–7.47)	7.38 (7.34–7.43)	0.027
PaO_2_/FiO_2_ ratio	327 (237–381)	227 (168–280)	0.004	253 (197–298)	160 (107–205)	< 0.001
PaCO_2_, mmHg	36 (30–43)	42 (36–49)	0.009	39 (33–43)	50 (38–57)	0.002
Lactate, mmol.L^−1^	1.2 (0.8–1.6)	1.3 (1.0–1.8)	0.254	0.9 (0.7–1.2)	1.1 (1.0–1.5)	0.015
Haemoglobin, g.dL^−1^	8.3 (7.8–10.4)	9.4 (8.4–10.7)	0.227	8.6 (7.9–10.7)	9.8 (8.8–11.5)	0.073
Delta haemoglobin, %	–	–		0 (-5–5)	5 (1–9)	0.013
Protein, g.L^−1^	55 (48–59)	60 (54–65)	0.021	53 (49–59)	64 (58–69)	0.001
Delta protein, %	–	–		4 (0–5)	7 (2–9)	0.014
Cardiac function						
LEVF, %	60 (50–60)	60 (50–60)	0.319	60 (50–60)	60 (50–60)	0.321
CI, L.min^−1^.m^−2^	3.1 (2.6–3.9)	3.6 (2.9–4.2)	0.264	3.6 (2.8–4.1)	4.3 (3.6–4.9)	0.167
e’ wave, cm.s^−1^	11 (9–12)	10 (9–12)	0.904	10 (9–13)	11 (9–13)	0.939
E/A ratio	0.8 (0.7–1.1)	1.0 (0.8–1.4)	0.177	0.9 (0.7–0.9)	1.1 (0.9–1.3)	0.001
E/e’ ratio	7.1 (5.3–8.6)	8.1 (6.6–10.5)	0.092	7.4 (5.3–9.2)	9.3 (7.2–1.28)	0.022
E/e’ > 14, *n* (%)	2 (10)	1 (3)		1 (10)	5 (19)	
DTE, ms	175 (142–210)	172 (137–201)	0.967	169 (142–228)	157 (140–200)	0.329
Systolic PAP, mmHg	23 (20–28)	27 (20–35)	0.255	24 (21–36)	30 (21–53)	0.165
TAPSE, cm	2.3 (1.9–2.6)	2.1 (1.9–2.7)	0.988	2.3 (2.1–2.7)	2.3 (1.9–2.9)	0.839
S wave, cm s^−1^	15.8 (13.7–18.7)	16 (14.0–21.1)	0.621	17.4 (14.1–20.1)	17.0 (15.6–20.5)	0.829
Diaphragm function						
Ptr,stim, cmH_2_O	6.8 (3.4–8.8)	4.1 (2.7–7.9)	0.098	6.0 (3.8–8.3)	4.3 (2.2–6.9)	0.192
Lung aeration						
Lung consolidation, *n*	2 (1–4)	4 (0–10)	0.026	2 (0–1)	6 (0–10)	0.025
LUS	10 (3–19)	17 (4–33)	0.123	9 (3–20)	20 (5–33)	0.060
Posterior LUS	7 (2–12)	10 (1–17)	0.377	6 (2–14)	11 (4–17)	0.112

SBT, spontaneous breathing trial; ABP, arterial blood pressure; LEVF, left ventricular ejection fraction; CI, cardiac index; E/A, early (E) over late (A) diastolic wave velocity ratio; E/e’, E wave over tissue Doppler early (e’) wave velocities at the lateral mitral valve annulus; DTE, deceleration time of the E wave; TAPSE, tricuspid annular plane systolic excursion (TAPSE); PAP, pulmonary artery pressure; LUS, lung ultrasound score

### Impact of diaphragm dysfunction on weaning-induced lung aeration loss

The lung ultrasound score was significantly higher in patients with diaphragm dysfunction compared to those without diaphragm dysfunction (21 (7–33) vs. 6 (0–15), *p* = 0.003). There was a moderate correlation between lung ultrasound score and Ptr,stim (*r* = −0.384 [95% CI −0.602 to–0.113, *p* = 0.005]).

## Discussion

This study shows that, in patients with difficult weaning: (1) diaphragm dysfunction and weaning-induced pulmonary oedema were frequent, both individually and combined and (2) lung ultrasound loss was associated with diaphragm dysfunction and spontaneous breathing trial failure.

### Coexistence of weaning-induced pulmonary oedema and diaphragm dysfunction

Herein, we report a high rate of spontaneous breathing trial failure that is purposely explained by the specific combination of inclusion criteria that we applied: duration of mechanical ventilation longer than 48 h and failure to the first spontaneous breathing trial. This prevalence is higher than that previously reported (60%) [1, 2, 27], but can presumably be explained by the selection of our patients. The definition of weaning-induced pulmonary oedema used in this study could also explain the high prevalence, as we opted to standardize classification of patients based on objective criteria (echocardiography and/or biomarkers) rather than classify patients based on expert consensus [1, 2], which may be subject to bias. We also used a challenging weaning trial that is likely to have markedly influenced the intrathoracic pressure [29]. In addition, it is likely that the early spontaneous breathing trial strategy relying on the presence of “readiness-to-wean criteria” rather on “readiness to extubate criteria” is partially responsible of the high prevalence of weaning-induced pulmonary oedema observed in our population.

Diaphragm dysfunction was present in 63% of our patients before starting the spontaneous breathing trial and appeared to be particularly severe (median Ptr,stim was 4.7 cmH_2_O). Of note, diaphragm dysfunction was defined by a lower Ptr,stim-threshold (7 cmH_2_O) than the classical definition of diaphragm dysfunction (11 cmH_2_O) [3, 4]. This definition was based on previous studies demonstrating that a threshold of 11 cmH_2_O is likely to over-diagnose diaphragm dysfunction in the ICU setting [21, 23].

**Fig. 4:**
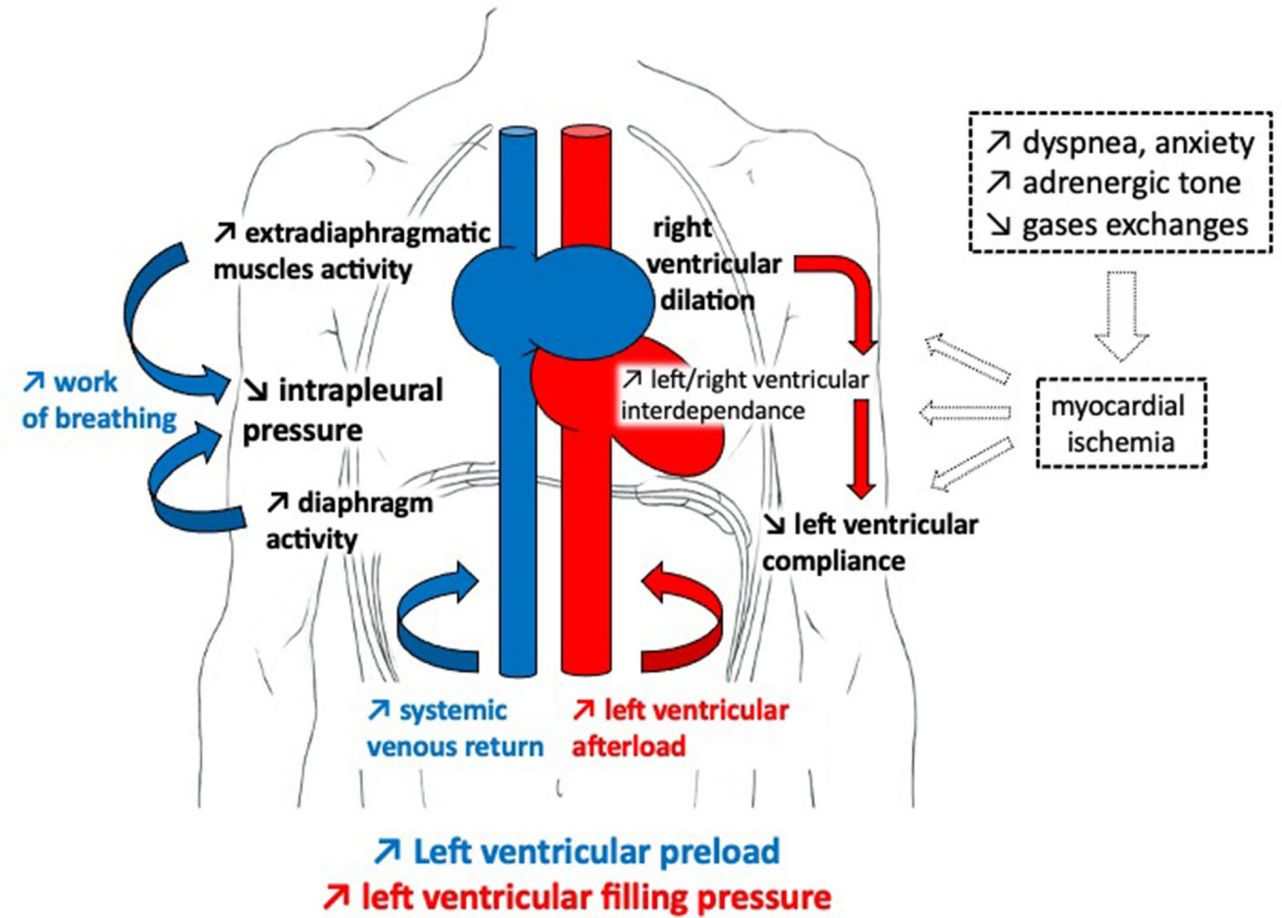
Main pathophysiological mechanisms involved in the occurrence of weaning-induced pulmonary oedema. The decrease is intrathoracic pressure is mediated through the contraction of the diaphragm and extradiaphragmatic respiratory muscles. It may lead to an increase in systemic venous return and cardiac preload. The decrease in intrathoracic pressure is also responsible for an increase in left ventricular afterload. The increase in right ventricular preload could induce a right ventricular dilation that can lead to ventricles interdependence and impaired left ventricular compliance potentially exacerbated by myocardial ischaemia

Large negative swings in intrathoracic pressure during a spontaneous breathing trial tend to increase the venous return pressure gradient, the central blood volume and the left ventricular filling pressure (Fig. 4). They also represent an increase in left ventricular afterload. These phenomena can contribute to weaning-induced pulmonary oedema [13]. Of note, diaphragm contractions are powerful drivers of venous return insofar as they simultaneously increase abdominal pressure while decreasing intrathoracic pressure, hence leading to an increased abdomino-thoracic gradient [10, 11]. As a result, diaphragm dysfunction may limit the occurrence of weaning-induced pulmonary oedema through a reduction in venous return and in inspiratory-related increases in left-ventricular afterload. By contrast, diaphragm dysfunction appeared to frequently coexist with weaning-induced pulmonary oedema. One possible explanation for this observation lies in the fact that in the presence of diaphragm dysfunction, extradiaphragmatic inspiratory muscles are recruited to generate large negative pleural pressure swings [30, 31]. This should cancel the possibly “positive” haemodynamic effects of diaphragm dysfunction through both an increased venous return and an increased left ventricular afterload [12, 32].

### Effect of diaphragm dysfunction on lung aeration loss

Excessive lung aeration loss at the time of the spontaneous breathing trial is associated with weaning failure [15, 33]. There are many causes for lung aeration loss, including alterations in lung compliance/resistance, ventilation/perfusion mismatch, atelectasis and pulmonary oedema. Respiratory muscle weakness has also been suggested to play a role in lung aeration loss during weaning [15], but has never been investigated. Studies performed in the operating room have demonstrated atelectasis induced by diaphragm paralysis [7–9]. Spontaneous ventilation results in preferential distribution of volume to dependent (posterior) lung regions, whereas controlled mechanical ventilation induces preferential ventilation of non-dependent (anterior) lung regions in anaesthetized and paralyzed subjects. A possible explanation for this shift in the ventilation pattern would be the effect of anaesthesia on regional lung compliance and changes in regional chest wall mechanics due to the paralysis of respiratory muscles, especially the diaphragm [9]. Lung ultrasound is an interesting tool in this context since it can show the occurrence of B lines that are mainly generated by weaning-induced pulmonary oedema [27, 28]. Our findings show that patients with diaphragm dysfunction had a significantly higher lung ultrasound score (21 vs. 6), suggesting a greater lung aeration loss compared to patients without diaphragm dysfunction. Importantly, the lung ultrasound score was significantly higher (the lung aeration loss was therefore greater) in patients who failed the spontaneous breathing trial and who had diaphragm dysfunction, whereas patients without diaphragm dysfunction who failed the spontaneous breathing trial had a low lung ultrasound score and consequently preserved lung aeration. Nevertheless, the high frequency of loss of aeration may be partially explain by the strategy of early spontaneous breathing trial performed during the ICU stay, especially when considering the substantial proportion of patients intubated for coma.

### Clinical implications

Our findings may have important clinical implications, since they highlight the fact that the presence of diaphragm dysfunction may not prevent the development of weaning-induced pulmonary oedema. It is noteworthy that a significant proportion of patients (up to 44%) can be successfully extubated despite diaphragm dysfunction [4] and a recent study found that diaphragm dysfunction was equally present in patients with extubation success and extubation failure [34]. In addition, a study reported that long-term survival was not influenced by the presence of diaphragm dysfunction at the time of weaning [35].

The main message for clinicians is that, despite the presence of diaphragm dysfunction present before starting a spontaneous breathing trial, it is still important to investigate other possible causes of spontaneous breathing trial failure, including weaning-induced pulmonary oedema, which is the commonest and easiest to manage cause. Importantly, in case of weaning-induced pulmonary oedema associated with diaphragm dysfunction, treatment of weaning-induced pulmonary oedema may facilitate weaning by improving the respiratory load/capacity balance. Since our population was selected early during the weaning phase at the time where patients were deemed ready to undergo a spontaneous breathing trial but not extubation, these clinical implications warrant further studies. Another important result of our study is the association between diaphragm dysfunction and lung atelectasis. This may be a useful finding, as the lung ultrasound score could be used as an endpoint of diaphragm function improvement in the context of future clinical trials.

## Limitations

This study has several limitations. First, neither intrathoracic pressure nor abdominal pressure was monitored during the spontaneous breathing trial. Consequently, no data can be provided concerning the level of intrathoracic pressure generated by respiratory muscles. Measuring the transdiaphragmatic pressure that is the result of the contraction of the diaphragm would have allowed to better elucidate the influence of the diaphragm function on cardiac function during weaning. Second, the diagnosis of weaning-induced pulmonary oedema was based on laboratory and echocardiographic criteria and not based on the reference method, i.e. measurement of pulmonary artery occlusion pressure elevation. However, current practices are based on alternatives to pulmonary artery catheter to diagnose weaning-induced pulmonary oedema, which is now considered to be an invasive technique at the time of weaning [13]. Third, the fluid balance was only obtained during the last 24 h preceding the spontaneous breathing trial. Fourth, the occurrence of mitral regurgitation was evaluated at baseline, but not at the end of the spontaneous breathing trial.

## Conclusion

Diaphragm dysfunction and weaning-induced pulmonary oedema are frequently associated in difficult-to-wean patients and diaphragm dysfunction is associated with lung aeration loss. This study suggests that the presence of diaphragm dysfunction in difficult-to-wean patients may encourage physicians to investigate the presence of weaning-induced pulmonary oedema. It may help to accelerate the weaning process by improving the respiratory load/capacity balance, particularly in case of diaphragm dysfunction.

## Data Availability

The datasets used and/or analysed during the current study are available from the corresponding author on reasonable request.
